# Flowering after disaster: Early Danian buckthorn (Rhamnaceae) flowers and leaves from Patagonia

**DOI:** 10.1371/journal.pone.0176164

**Published:** 2017-05-10

**Authors:** Nathan A. Jud, Maria A. Gandolfo, Ari Iglesias, Peter Wilf

**Affiliations:** 1 L. H. Bailey Hortorium, Plant Biology Section, School of Integrative Plant Science, Cornell University, Ithaca, New York, United States of America; 2 Universidad Nacional del Comahue, Instituto de Investigaciones en Biodiversidad y Ambiente INIBIOMA-CONICET, San Carlos de Bariloche, Rio Negro, Argentina; 3 Department of Geosciences, Pennsylvania State University, University Park, Pennsylvania, United States of America; Institute of Botany, CHINA

## Abstract

Southern-Hemisphere terrestrial communities from the early Paleocene are poorly known, but recent work on Danian plant fossils from the Salamanca Formation in Chubut Province, Argentina are providing critical data on earliest Paleocene floras. The fossils described here come from a site in the Salamanca Formation dating to ca. 1 million years or less after the end-Cretaceous extinction event; they are the first fossil flowers reported from the Danian of South America, and possible the entire Southern Hemisphere. They are compressions and impressions in flat-laminated light gray shale, and they belong to the family Rhamnaceae (buckthorns). Flowers of *Notiantha grandensis* gen. et sp. nov. are pentamerous, with distinctly keeled calyx lobes projecting from the hypanthium, clawed and cucullate emarginate petals, antepetalous stamens, and a pentagonal floral disk that fills the hypanthium. Their phylogenetic position was evaluated using a molecular scaffold approach combined with morphological data. Results indicate that the flowers are most like those of extant ziziphoid Rhamnaceae. The associated leaves, assigned to *Suessenia grandensis* gen. et sp. nov. are simple and ovate, with serrate margins and three acrodromous basal veins. They conform to the distinctive leaves of some extant Rhamnaceae in the ziziphoid and ampelozizyphoid clades. These fossils provide the first unequivocal megafossil evidence of Rhamnaceae in the Southern Hemisphere, demonstrating that Rhamnaceae expanded beyond the tropics by the earliest Paleocene. Given previous reports of rhamnaceous pollen in the late Paleogene and Neogene of Antarctica and southern Australia, this new occurrence increases the possibility of high-latitude dispersal of this family between South America and Australia via Antarctica during the Cenozoic.

## Introduction

The Salamanca Formation is an estuarine unit in the San Jorge Basin of southern Argentina that yields well-preserved, well-dated fossils from the early Paleocene. Studies of these fossils are providing new data on plant and animal diversity following the end-Cretaceous extinction event [1–19]. Here, we report the first fossil flowers from an early Danian (~65Ma) assemblage in the Southern Hemisphere and show that they are attributable to Rhamnaceae. Extant Rhamnaceae Juss. comprise 54 genera and over 900 species of shrubs, trees, lianas, and perennial herbs that are easily identified for their unusual combination of floral characters [20–23]. Traditionally, the family was subdivided into five tribes differentiated based on fruit types [24,25]; however, recent molecular phylogenetic studies suggest that these were not natural groups [26]. Instead, the family is now divided into 11 tribes that are distinguished by combinations of vegetative and reproductive character states, with a handful of genera still unplaced at the tribal level [21–23,26,27]. The tribes and unplaced genera belong to three major clades that are informally known as the rhamnoids, ziziphoids, and ampelozizyphoids; however, morphological synapomorphies for these three groups have not been identified so far [26]. Despite recent advances in understanding the systematics of living Rhamnaceae, many aspects of their early evolution and biogeographic history remain unclear [22,23,28–30].

**Fig 1:**
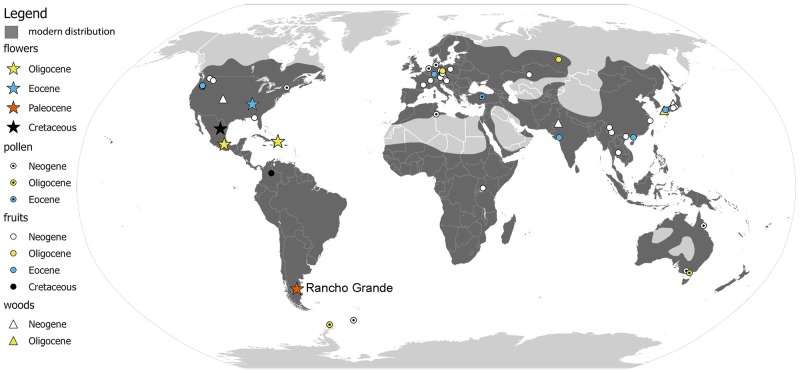
World map showing the distribution of modern and fossil Rhamnaceae. Gray area indicates the distribution of extant Rhamnaceae. The shapes correspond to different fossilized plant organs and the colors represent different ages. The fossil flowers and leaves described here were collected from the Rancho Grande site in Chubut Argentina. Details of all occurrences shown here are listed in Table 1. The base map was created with Natural Earth Dataset.

In the last decade, the fossil record of Rhamnaceae has grown significantly (Fig 1; Table 1), and several occurrences have confirmed at least a Late Cretaceous origin for the family [28,31]. Fossil remains assigned to extant genera have been reported from Eocene and younger deposits, including the distinctive fruits of *Paliurus* Mill. [32–41], *Berchemia* Neck. ex DC. [42], and *Ventilago* Gaertn. [31,43], the wood and leaves of *Hovenia* Thunb. [37,44–46]; and the distinctive leaves of *Ceanothus* L. [38,47–50] and *Colubrina* Rich. ex Brongn. [51,52]. These fossils provide minimum age estimates for the diversification of crown-group Rhamnaceae, and several have been used to calibrate trees in recent molecular phylogenetic analyses [29,53,54].

**Table 1:** Summary of fossil Rhamnaceae. Occurrences of fossils attributed to Rhamnaceae, excluding those based on leaf compressions alone.

Taxon	Organ	Latitude	Longitude	Age	Reference
*Coahuilanthus belindae* Calvillo-Canadell et Cevallos-Ferriz	flower	25.50	-101.30	Cretaceous	[31]
*Archaeopaliurus boyacensis* Correa, Jaramillo, Manchester et Gutierrez	fruit, leaves	5.90	-72.80	Cretaceous	[28]
*Notiantha grandensis* gen. et sp. nov.	flower	-45.55	-68.25	Paleocene	this study
*Solanites pusillus* Berry	flower	36.42	-88.35	Eocene	[30,103]
*Berchemia eocenica* Collinson, Manchester et Wilde	fruit	49.90	8.75	Eocene	[42]
*Paliurus favonii* Unger	fruit	21.80	110.90	Eocene	[41]
*Paliurus clarnensis* Burge et Manchester	fruit	44.75	-120.60	Eocene	[38]
*Paliurus ubensis* Huzioka et Takahasi	fruit	33.95	131.25	Eocene	[148]
*Paliurus* sp.	fruit	44.18	-120.20	Eocene	[37]
*Paliurus clarnensis* Burge et Manchester	fruit	44.73	-120.40	Eocene	[38]
*Paliurus clarnensis* Burge et Manchester	fruit	44.59	-120.26	Eocene	[38]
*Paliurus clarnensis* Burge et Manchester	fruit	44.70	-120.42	Eocene	[38]
*Paliurus clarnensis* Burge et Manchester	fruit	44.74	-120.47	Eocene	[38]
*Ziziphus eocenicus* Singh et al. 2010	fruit	21.40	73.12	Eocene	[149]
*Nahinda axamilpensis* Calvillo-Canadell et Cevallos Ferriz	flower	18.60	-97.90	Oligocene	[31]
*Distigouania irregularis* Chambers et Poinar	flower	19.80	-70.75	Oligocene	[104]
*Comopellis presbya* Chambers et Poinar	flower	19.80	-70.75	Oligocene	[105]
*Ventilago engoto* Calvillo-Canadell et Cevallos-Ferriz	fruit	18.60	-97.90	Oligocene	[31]
*Hovenia palaeodulcis* Suzuki	wood	33.79	130.46	Oligocene	[45]
*Paliurus sibirica* Dorofeev	fruit	56.80	84.49	Oligocene	[150]
*Paliurus sibirica* Dorofeev	fruit	51.47	13.62	Oligocene	[150]
*Paliurus favonii* Unger	fruit	22.15	107.02	Miocene	[40]
*Paliurus microcarpa* Li	fruit	29.15	121.25	Miocene	[39]
*Ventillago lincangensis* Liu et Xie	fruit	23.90	100.00	Miocene	[43]
*Paliurus tiliaefolius* (Unger] Bŭžek	thorny twigs & leaves	50.55	13.77	Miocene	[151]
*Hovenia cf dulcis* Suzuki	wood	36.57	136.60	Miocene	[44]
*Hovenia palaeodulcis* Suzuki	wood	38.90	-105.29	Miocene	[46]
*Palurus favonii* Unger	fruit	51.63	12.35	Miocene	[152]
*Palurus favonii* Unger	fruit	50.55	13.76	Miocene	[153]
*Palurus favonii* Unger	fruit	50.55	13.76	Miocene	[153]
*Palurus favonii* Unger	fruit	50.55	13.76	Miocene	[154]
*Paliurus thurmanii* Heer	fruit	50.55	13.76	Miocene	[154]
*Paliurus thurmanii* Heer	fruit	48.43	12.37	Miocene	[155]
*Paliurus ovoideus* Goeppert	fruit	51.28	14.09	Miocene	[156]
*Palurus favonii* Unger	fruit	47.47	15.28	Miocene	[157]
*Palurus favonii* Unger	fruit	47.47	15.28	Miocene	[157]
*Paliurus thurmanii* Heer	fruit	47.07	6.70	Miocene	[158]
*Paliurus tiliaefolius* Unger	fruit	50.36	13.25	Miocene	[34]
*Paliurus tiliaefolius* Unger	fruit	50.36	13.25	Miocene	[34]
*Paliurus aff*. *aculeatus* Lam.	fruit	52.21	18.25	Miocene	[159]
*Palurus favonii* Unger	fruit	46.94	15.79	Miocene	[157]
*Paliurus fricii* Brabenec	fruit	50.26	13.57	Miocene	[160]
*Paliurus zaporogensis* Krysht.	fruit	49.68	63.43	Miocene	[35]
*Paliurus* sp.	fruit	26.12	99.33	Miocene	[161]
*Paliurus protonipponicus* Suzuki	fruit	35.22	137.08	Miocene	[162]
*Paliurus hesperius* Berry	fruit	47.94	-119.00	Miocene	[163]
*Paliurus hesperius* Berry	fruit	47.66	-117.43	Miocene	[164]
*Paliurus* sp.	fruit	47.94	-119.00	Miocene	[165]
*Paliurus* sp.	fruit	47.01	-116.25	Miocene	[166]
*Paliurus* sp.	fruit	30.47	-84.99	Miocene	[167,168]
*Ziziphus* sp.	wood	28.18	73.30	Pliocene	[135]
*Paliurus nipponicus* Miki	fruit	34.64	135.03	Pliocene	[33]
*Paliurus aff*. *hemsleyanus*	fruit	45.05	2.72	Pliocene	[169]
*Ziziphus khoksungensis* Grote	fruit	15.05	102.12	Pleistocene	[170]
*Paliurus nipponicus* Miki	fruit	34.82	135.82	Pleistocene	[32]
Rhamnaceae	pollen	43.83	-73.05	Neogene	[171,172]
Rhamnaceae	pollen	52.52	5.5	Neogene	[173]
Rhamnaceae	pollen	54.2	9.7	Neogene	[174]
Rhamnaceae	pollen	18.53	-98.7	Oligocene	[175]
Rhamnaceae	pollen	39.75	34.84	Eocene	[176]
Rhamnaceae	pollen	-60	42	Neogene	[128]
Rhamnaceae	pollen	32	10	Neogene	[177]
Rhamnaceae	pollen	-17.36	145.69	Neogene	[126]
Rhamnaceae	pollen	-37.35	144.13	Neogene	[130]
Rhamnaceae	pollen	-62.15	-58.45	Oligocene	[127]
Rhamnaceae	pollen	-38.25	146.38	Oligocene	[128]
*Berchemia pseudodiscolor* Chesters	fruit	-0.4	34.17	Miocene	[178, 179, 180]
*Ziziphus miocenicus* Chesters	fruit	-0.4	34.17	Miocene	[178]
*Ziziphus rusingensis* Chesters	fruit	-0.4	34.17	Miocene	[178, 179, 180]

Whereas some authors have suggested a Laurasian origin for the Rhamnaceae family [55], Richardson et al. [53] concluded that a Gondwanan origin during the Paleogene is more parsimonious for the ziziphoid and the ampelozizyphoid clades. Most recently, Onstein et al. [26,56] concluded that crown-group Rhamnaceae began to diversify in the tropical rainforest biome during the Cretaceous, rather than the Paleogene [53,57], but also that much of the modern richness can be attributed to relatively recent (latest Paleogene to Neogene) diversification in Mediterranean-type ecosystems.

The fossil flowers reported here are adpressions, and they have a combination of character states similar to members of the ziziphoid clade and the tribe Paliureae Reissek ex Endl, but they do not match any extant genus. Although not in organic connection, the flowers were found in association with ziziphoid leaves, also described here. We use phylogenetic analysis to determine the relationship of these fossils to living members of the family and to test biogeographic hypotheses, including the idea that the Rhamnaceae originated in Laurasia.

### Geologic setting

The Salamanca Formation crops out in the San Jorge Basin in southern Chubut and northern Santa Cruz provinces, Argentina, overlying the Cretaceous Chubut Group and underlying the Paleocene and Eocene Río Chico Group [10,17, 18, 58–61]. The formation yields abundant plant remains [2,3,8,9,15,16,19,62–65] as well as fossils of invertebrates [10,65–71], marine macrofaunas [72–74], reptiles [75–77], and mammals [1,4–7,11–14,78,79].

The fossils described here were collected from the Rancho Grande locality in Chubut, a single fossil quarry exposed along the banks of the Río Chico in the lower Salamanca Formation [17,18]. All necessary permits were obtained for the described study, which complied with all relevant regulations. The age of the Rancho Grande site is constrained to geomagnetic polarity chron C29n, or 65.58–64.86 Ma (early Danian) on the 2012 Geomagnetic Polarity Timescale [17,18,80]. The formation consists primarily of estuarine to shallow marine deposits, and the fossils were found in flat-laminated beds of very-fine sandstone to siltstone. The Rancho Grande beds were deposited in a tidal estuary near the seaward limit of tidal influence [18]. Abundant angiosperm leaves, delicate flowers, and leafy herbaceous shoots characterize the assemblage. The presence of a diverse marine fauna including brittle stars, a benthic foraminifer, and bivalves in the same bedding planes suggests significant transport of the plant material from the original site of growth [39,46]

Palynological analysis of Danian deposits in northern Chubut Province revealed low floral diversity after the end-Cretaceous mass extinction, followed by a rapid recovery [63]. Recent analysis of palynomorphs collected from the same temporal interval of the Salamanca Fm. as the fossil flowers and leaves described here (C29n) found that 50% of all pollen types are angiosperms, whereas gymnosperms accounted for only ~13% of total richness; however, *Classopollis* pollen, representing the extinct conifer family Cheirolepidiaceae, is the most abundant palynomorph in all samples [17]. Wood assemblages from the Salamanca Fm. are dominated by conifers, but the presence of fossil angiosperm woods indicate that they were also part of the canopy [8,9,65]. The co-occurrence of palms [2,3,16,64,65,81], dicot woods with indistinct growth rings [9], and alligatorids [76,77], indicates temperature remained above freezing year-round. The results of leaf physiognomic analyses [15,82,83] indicate that the climate in the San Jorge Basin during the early Paleocene was warm subtropical.

## Materials and methods

The fossil specimens are housed in the Paleobotanical collection of the Museo Paleontológico Egidio Feruglio (MPEF-Pb), Trelew, Chubut Province, Argentina, under these numbers: MPEF-Pb 8548a&b, MPEF-Pb 8549, MPEF-Pb 8551 (flowers), MPEF-Pb 8552, MPEF-Pb 8553, MPEF-Pb 8555, MPEF-Pb 8560, MPEF-Pb 8563 (leaves). The fossil flower specimens were prepared using standard degauging techniques, whereas the leaves required minimal preparation. Images of macroscopic features were captured with a Canon EOS 7D DSLR Camera, and microscopic details were photographed with a Nikon DS Fi1 camera mounted on a Nikon SMZ1000 stereoscope at the Museo Paleontológico Egidio Feruglio. Epifluorescence microscopy was used to check for the presence of pollen grains in the anthers. Images were processed with Adobe Photoshop (San Jose, California, USA). The fossils were compared with extant Rhamnaceae specimens obtained from the LH Bailey Hortorium Herbarium (BH), Department of Plant Biology, Cornell University, Ithaca, NY, USA, the U.S. National Herbarium (US), Smithsonian National Museum of Natural History, Washington DC, USA, the National Cleared Leaf Collection (NCLC-H) Smithsonian National Museum of Natural History, Washington DC, USA, and the University of Florida Herbarium (FLAS), University of Florida, Gainesville, FL, USA (S1 Table). Terminology for description of the leaves follows that of the Manual of Leaf Architecture [84].

To evaluate the phylogenetic affinities of the fossil flowers, and the plant concept based on both flowers and leaves, we assembled a new morphological matrix for Rhamnaceae modified from that of Calvillo-Canadell [85] as later published by Millán and Crepet [30]. We compared the floral characters with those included in the studies by Aagesen [86], Richardson et al. [27], and Islam and Simmons [87]. Based on these comparisons, we made several changes to the original matrix of morphological characters. First, we modified several characters to so that there are fewer alternative states and so that additional fossil and modern material is easier to score, but also so that the results are not in conflict with previous analyses. Second, we excluded characters that are not preserved in any of the fossil flowers because they would not influence the optimal position of the fossil-taxon on the scaffold topology. Third, we added three characters related to pubescence and floral disk morphology because these features are preserved on the fossils. Fourth, we added five foliar characters. Fifth, we scored character data for seven additional extant genera *Sarcomphalus* R. Browne emend. Hauenschild, *Hovenia* (Paliureae), *Ventilago* (Ventilagineae), *Pomaderris* Labill. (Pomaderreae), *Noltea* Rchb. (Phyliceae), *Ampelozizyphus* Duckey (Ampelozizypheae), *Bathiorhamnus* Capuron (Bathiorhamneae), and *Helinus* E. May. ex Endl. (Gouanieae Reissek ex Endl.). Finally, we scored character data for the fossil flowers and leaves. The matrix comprises 25 taxa, 25 floral characters, and five leaf characters. The complete matrix is available online at the MorphoBank website (project P2506, Morphology of Rhamnaceae (flowers and leaves) [matrix 24392]; http://morphobank.org/permalink/?P24392). Nine of the 11 tribes of Rhamnaceae are represented in this new matrix, as opposed to four tribes in the matrix used by Millán and Crepet [30]. With the inclusion of *Sarcomphalus* (formerly new world *Ziziphus* Mill.) and *Hovenia*, the generic diversity of Paliureae is fully represented in this new dataset. Of the three monogeneric tribes in the ampelozizyphoid clade, *Ampelozizyphus* and *Bathiorhamnus* are included [88,89].

We analyzed the matrix using the molecular scaffold approach described by Springer et al. [90] to determine the most parsimonious position(s) first based on the fossil flowers alone, and then including the foliar characters. We used two different scaffolds to evaluated how sensitive the placement of the fossil is to tree topology. First, we constrained the tree searches such that the final topology is consistent with the relationships reported by Hauenschild et al. [23]. The Hauenschild et al. topology is based on sequence data from one chloroplast marker (*trnL-trnF*) and one nuclear marker (ITS) for more than 400 species. Then, we constrained the tree searches such that the final topology is consistent with the relationships reported by Onstein et al. [29]. The Onstein et al. topology is based sequence data from six chloroplast markers and one nuclear marker (ITS) for 280 species. All tree searches were implemented in the phylogenetic software TNT [91] using the parsimony ratchet [92]. We constrained the searches by appending a set of binary characters that define the scaffold topology and weighting them to 99%. All characters were unordered, and only the position of the new taxon was free to vary. In the analyses, 10 sets of 200 iterations using a 10% perturbation of characters were used for the ratchet analyses, and default values for drift, sectorial search, and tree fusion were retained.

### Nomenclature

The electronic version of this article in Portable Document Format (PDF) in a work with an ISSN or ISBN will represent a published work according to the International Code of Nomenclature for algae, fungi, and plants, and hence the new names contained in the electronic publication of a PLOS article are effectively published under that Code from the electronic edition alone, so there is no longer any requirement to provide printed copies.

New names contained in this work have been submitted to IFPNI, from where they will be made available to the Global Names Index. The LSID IFPNI codes can be resolved and the associated information viewed through any standard web browser by appending the LSID contained in this publication to the prefix http://fossilplants.info/. The LSID for this publication is: http://fossilplants.info/publications/4B0FD041-D9CE-446E-B866-D201D710F412. The online version of this work is archived and available from the Dryad Digital Repository.

## Results

### Systematics

**Order**: Rosales Bercht. & J. Presl 1820

**Family**: Rhamnaceae Jussieu 1789

**Genus**: *Notiantha* Jud, Gandolfo, Iglesias & Wilf, gen. nov.

**Fig 2:**
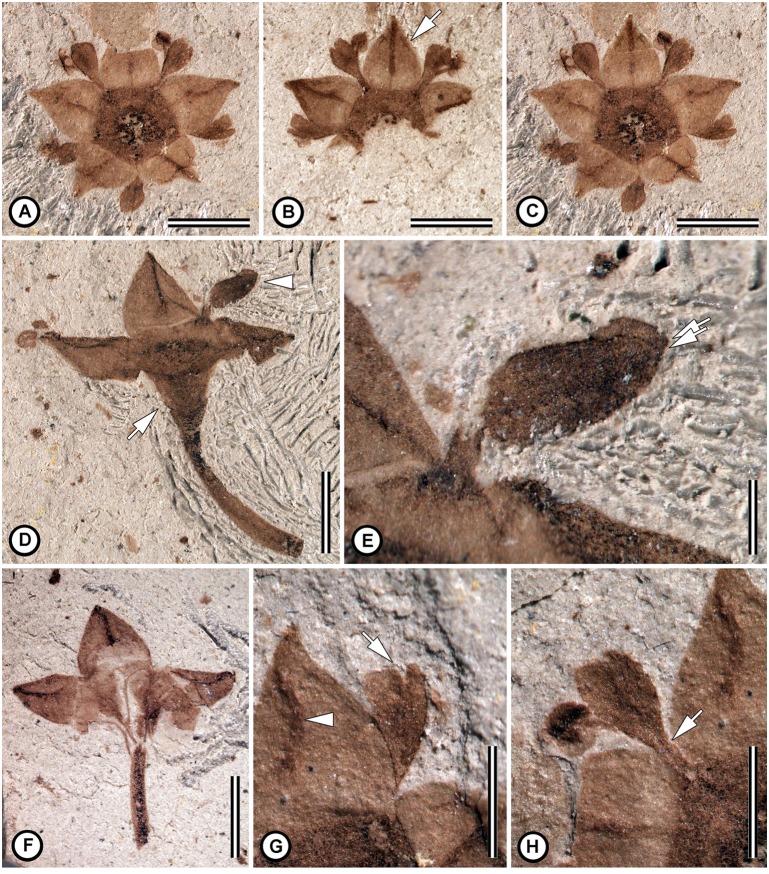
*Notiantha grandensis* Jud, Gandolfo, Iglesias & Wilf, gen et sp. nov. (A) Flower in transverse view showing pentamerous structure, sepals triangular with a distinct keel, cucullate petals alternating with sepals, stamens antepetalous, and floral disk surrounding a coalified gynoecium. MPEF-Pb 8548a. (B) Counterpart of specimen in ‘A’ showing a sepal with a central keel and two marginal veins converging toward the apex (at arrow) MPEF-Pb 8548b. (C) Composite digital illustration of the flower created from ‘A’ and ‘B’. (D) Flower in longitudinal view showing slender pedicel, floral cup (at arrow), three preserved sepals, and a cucullate petal (at arrowhead). MPEF-Pb 8549. (E) Close-up of the petal in ‘D’ showing clawed structure and the longitudinally folded distal portion of the petal; the overlapping lobes are marked with arrows. MPEF-Pb 8549. (F) Flower in longitudinal view showing slender pedicel and three sepals. MPEF-Pb 8551. (G) Close-up of flower in ‘A’ showing the keeled sepal (at arrowhead), and the notched petal apex (at arrow). MPEF-Pb 8548a. (H) Close-up of flower in ‘A’ showing an anther opposite a petal, and a line suggesting where the anther filament adnate to the petal at its base (at arrow). MPEF-Pb 8548a. Scale bars: A-D, F = 2 mm; E = 0.5 mm; G, H = 1 mm.

**Type species**: *Notiantha grandensis* Jud, Gandolfo, Iglesias & Wilf, sp. nov. (Fig 2).

**Generic diagnosis**: Pedicellate, pentamerous, actinomorphic, perfect flowers; hypanthium obconical, glabrous; calyx lobes five, lobes deltoid to ovate, keeled, inserted at the margin of the hypanthium; petals five, short-clawed, cucullate, curved, and equal in length to the calyx lobes, petal apex emarginate; stamens epipetalous, anthers dorsifixed and versatile; pentagonal floral disk filling the hypanthium.

**Etymology**: From the Greek nótios for southern, and anthos for flower.

**Species**: *Notiantha grandensis* Jud, Gandolfo, Iglesias & Wilf, sp. nov. Fig 2A–2H.

**Holotype**: MPEF-Pb 8548a,b.

**Paratypes**: MPEF-Pb 8549, MPEF-Pb 8551.

**Repository**: Museo Paleontológico Egidio Feruglio Paleobotany Collection (MPEF-Pb), Trelew City, Chubut, Argentina.

**Type Locality**: Rancho Grande, Chubut, Argentina.

**Stratigraphic position**: Lower Salamanca Formation.

**Age**: Paleocene, early Danian, geomagnetic polarity chron C29n (65.58–64.86 Ma).

**Etymology of specific epithet**: from the Rancho Grande locality.

**Species diagnosis**: as for the genus *Notiantha*.

**Description**: The flowers are pedicellate, pentamerous, actinomorphic, and perfect, 5–7 mm diameter (Fig 2A–2C) with a gamosepalous, obconical floral cup. The pedicel is slender, 2.5–4 mm long and 0.6 mm across (Fig 2D and 2F). The perianth is composed of calyx and corolla that have whorled phyllotaxy. The calyx lobes (sepals) are triangular to slightly ovate with acute and straight to slightly acuminate apex (Fig 2A–2C and 2F–2H), and they are 1.4–1.6 mm wide and 1.5–1.7 mm long. A distinct adaxial, longitudinal keel (Fig 2A–2D and 2F–2H) and two converging marginal veins are visible on each sepal (Fig 2B). The corolla is composed of short-clawed, cucullate petals (Fig 2A–2E) with an apical notch (i.e., emarginate apex; Fig 2G); they are either open (Fig 2G and 2H) or conduplicate (Fig 2E) and alternating with sepals (Fig 2A–2C); petals are 1.4 mm long, 0.1 mm wide at base and 0.6 mm at the widest part. The androecium has five antepetalous stamens (i.e. obhaplostemonous) (Fig 2A–2C), which are adnate to the petals at the base (Fig 2H); the filaments are slender, c. 1.2 mm long, and the anthers are dorsifixed and versatile and seem to have four microsporangia (Fig 2H). Pollen grains were not detected in the anthers. The gynoecium is poorly preserved, and the number of carpels and stylodia is unknown. In transverse view, the pentagonal area that is 1.9 mm across, darker than the sepals, and surrounds the coalified gynoecium is interpreted as a floral disk. The floral disk surrounds and covers much of the coalified gynoecium, suggesting that it is either semi-inferior or inferior.

**Genus**: *Suessenia* Jud, Gandolfo, Iglesias & Wilf, gen. nov.

**Fig 3:**
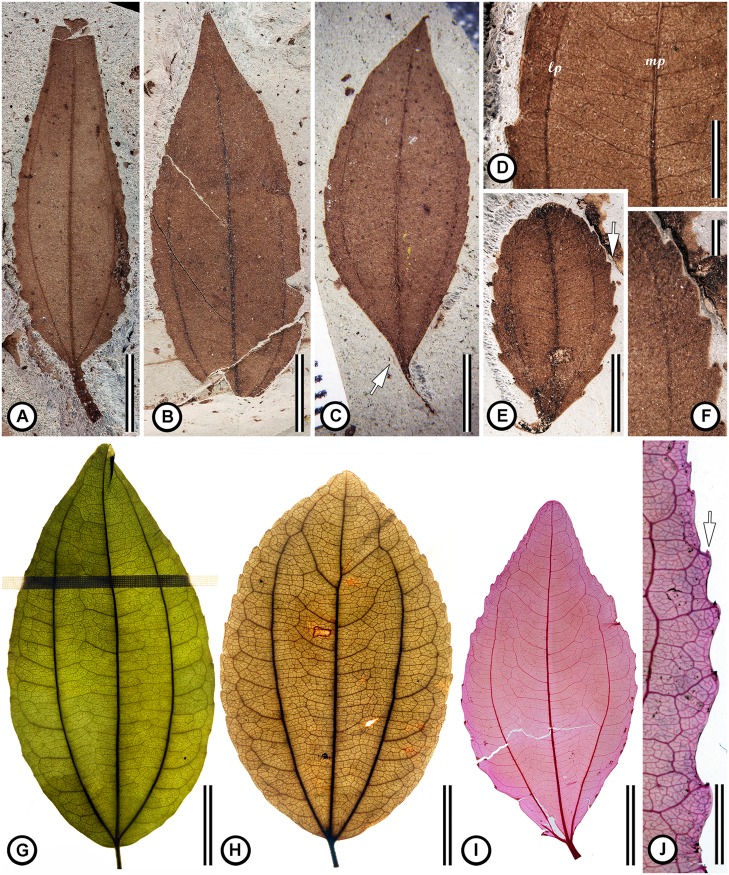
Comparison of fossil (A-F) and modern Rhamnaceae leaves (G-J). (A) *S*. *grandensis* MPEF-Pb 8553 showing overall shape, stout petiole, acute base (at arrow), serrate margin, and acrodromous primary veins. (B) *S*. *grandensis* MPEF-Pb 8560 showing its shape, acute to attenuate apex (at arrow), serrate margin, and acrodromous primary veins. (C) *S*. *grandensis* MPEF-Pb 8555 showing overall shape, petiole, acute base, serrate margin, and acrodromous primary veins. (D) Close-up of the leaf blade (MPEF-Pb 8552) showing mixed percurrent epimedial tertiary veins running between the medial primary vein (mp) and the lateral primary vein (lp). Note that they form an acute angle to the medial primary vein. (E). *S*. *grandensis* MPEF 8563 overall shape, petiole, acute base, serrate margin, and acrodromous primary veins, and an asymmetric, obtuse apex. (F) Close-up of the margin in ‘E’ showing exterior tertiary veins that are looped or terminating at the margin; note the glandular tooth apex (at arrow). (G) Leaf of *Sarcomphalus saeri* (Pittier) Hauenschild US 2045934 showing ovate blade, petiole, rounded base, acute apex, serrate margin, three acrodromous primary veins, and alternate percurrent epimedial tertiary veins. (H) Leaf of *S*. *saeri* US 3554997 showing ovate blade, petiole, rounded base, acute apex, serrate margin with apically oriented teeth, three acrodromous primary veins, distal major secondary veins, and alternate percurrent epimedial tertiary veins. (I) Cleared leaf of *Ziziphus sativa* Gaertn. (junior synonym of *Z*. *jujuba* Miller) NCLC-H 1791 showing ovate to elliptic blade, acute base, acute apex, serrate margin with apically oriented teeth, three acrodromous primary veins. (J) Close-up of the leaf in ‘I’ showing the apically oriented glandular teeth. Note the similarity to ‘F.’ Scale bars: A, B, H = 10 mm; C, E = 5 mm; D = 3 mm; F, J = 2 mm; G = 15 mm; I = 40 mm.

**Type Species**: *Suessenia grandensis* Jud, Gandolfo, Iglesias & Wilf, sp. nov. (Fig 3).

**Generic Diagnosis**: Leaves simple, marginal petiolate; blade shape ovate to elliptic; base obtuse, rounded, symmetrical or slightly asymmetrical, apex acute or obtuse; margin unlobed, toothed; primary vein framework basal acrodromous, with three basal veins (rarely 5), agrophic veins absent or present; major secondary vein framework absent or semicraspedodromous distally; tertiary vein framework mixed percurrent; tooth spacing regular, teeth apically pointed, with indeterminate glandular tissue on the tooth apex.

**Etymology**: Named for K. Suessenguth in honor of his early work on the classification of Rhamnaceae.

**Species**: *Suessenia grandensis* Jud, Gandolfo, Iglesias & Wilf, sp. nov. Fig 3A–3F.

**Holotype**: MPEF-Pb 8553.

**Repository**: Museo Paleontológico Egidio Feruglio Paleobotany Collection (MPEF-Pb), Trelew City, Chubut, Argentina

**Type Locality**: Rancho Grande. Chubut, Argentina.

**Stratigraphic position**: Lower Salamanca Formation.

**Age**: Paleocene, early Danian, geomagnetic polarity chron C29n (65.58–64.86 Ma).

**Etymology of specific epithet**: from the Rancho Grande locality.

**Species diagnosis**: as for the genus *Suessenia*.

**Description**: Leaves simple, petiolate. Petiole stout, blade attachment marginal, laminar size microphyll. Laminar shape ovate to elliptic, with medial symmetry, length to width ratio 3:1 (7:3–4:1) (Fig 3A–3C). Apex acute, straight to acuminate, symmetrical (Fig 3B), base angle obtuse to acute, rounded to cuneate, insertion slightly asymmetrical (Fig 3A). Margin unlobed, serrate (Fig 3A–3F). Primary vein framework palmate acrodromous with three basal veins. Basal veins naked in some specimens (Fig 3C, at arrow). Major secondary veins not present, agrophic veins absent. Intercostal tertiary vein fabric (between the primary veins) mixed percurrent, angle of the percurrent tertiaries acute (Fig 3D). External tertiary veins supply the teeth (Fig 3F). Quaternary vein fabric irregular reticulate (Fig 3D). Tooth frequency decreasing distally, with one order, three teeth per cm (Fig 3A–3D). Sinus shape angular, tooth shape straight/retroflexed. Tooth apex pointed distally, with medial principal vein terminating at the tooth apex; each tooth with a gland on the apex (Fig 3E and 3F). Number of specimens examined: 31

### Phylogenetic analysis

**Fig 4:**
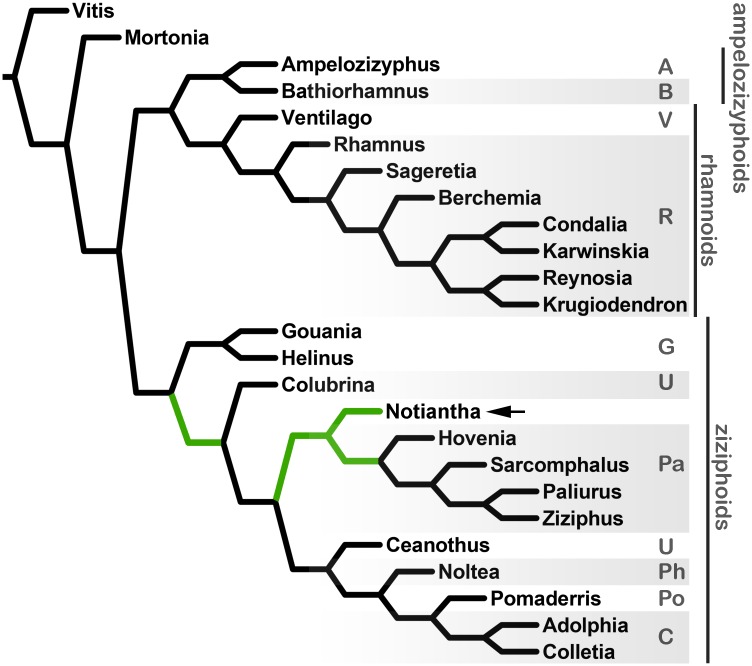
One of two equally most parsimonious trees based on floral characters and the topology of Hauenschild et al. [23] showing the position of *Notiantha* sister to the extant Paliureae [*Hovenia*+*Sarcomphalus*+*Paliurus*+*Ziziphus*] at arrow. The alternate most parsimonious position in the ziziphoid clade for the fossil flowers is colored in dark green. A = Ampelozizypheae, B = Bathiorhamneae, V = Ventilagineae, R = Rhamneae, Po = Pomaderreae, C = Colletieae, Ph = Phyliceae, U = unplaced genera at tribal level, G = Gouanieae, Pa = Paliureae.

The tree search using only floral characters and constrained by the topology of Hauenschild et al. [23], yielded two equally most-parsimonious trees: one with *Notiantha* deep in the ziziphoid clade and another sister to the extant Paliureae (Fig 4). The next tree search using the full 30 characters (from both flowers and leaves) resulted in five best trees. Three with *Notiantha* nested in the Paliureae, one with *Notiantha* sister to the Paliureae, and one with *Notiantha* sister to all ziziphoids except Gouanieae (S1 Fig). In all of the most-parsimonious trees obtained using the Hauenschild et al. [23] topology, the fossil-taxon was nested within the ziziphoid clade. The tree searches using the alternate topology reported by Onstein et al. yielded slightly differen results. The first analysis using only the floral characters for *Notiantha* resulted in eight most-parsimonious trees. One tree found *Notiantha* sister to the extant ampelozizyphoids, *Ampelozizyphus* and *Bathiorhamnus* (Fig 4), whereas all other trees placed *Notiantha* within the ziziphoid clade: either unplaced (e.g. sister to *Ceanothus*) or within the Paliureae (S2 Fig). Some trees in which the fossil is nested among the rhamnoids are only two steps longer than the optimal trees shown in (S2 Fig). The final tree search, using the full set of 30 floral and foliar characters, yielded four equally most-parsimonious trees. In one tree, the composite fossil-taxon is sister to the ampelozizyphoid clade, but in the other three trees, it is nested in the Paliureae (S3 Fig).

## Discussion

### Comparison with extant and fossil plants

#### Extant flowers

The flowers of *Notiantha grandensis* are readily assignable to Rhamnaceae because of their pentamerous structure, the obconical hypanthium with triangular keeled calyx lobes (sepals), clawed and cucullate petals, antepetalous stamens, and the floral disk [20,93,94]. Pentamerous flowers with antepetalous stamens are also found in Basellaceae Raf., Vitaceae Juss., Santalales Berchtold & J. Presl, and Gunneraceae Meisn. [95], but rhamnaceous flowers can be distinguished from among these when they have the combination of keeled sepals, clawed petals, filaments adnate to the petals, a hypanthium, or a fleshy floral disk.

As previously mentioned, there are eleven recognized tribes in Rhamnaceae, but several genera remain unplaced [26,27]. Many of the characters that have been identified as useful for distinguishing the tribes [21,27] are not preserved in the Patagonian fossils. The results of our phylogenetic analysis indicate that the fossil flowers are most like members of the ziziphoid clade, and the tribe Paliureae (Fig 4). The features supporting this relationship are the obconical floral cup, triangular to deltoid sepals each with a prominent keel, the floral disk adnate to the ovary filling the floral tube, the presence of short-clawed, cucullate petals with an apical notch, and a semi-inferior to superior ovary. By contrast, other tribes vary in the thickness and position of the floral disks, the shape of the hypanthium, and/or shape of the sepals. *Notiantha* differs from most extant members of Paliureae because the petals are apically emarginate and the floral disk is unlobed. In extant Paliureae, notched petals are rare, and they also occur outside of Paliureae in *Condalia* Cav., *Karwinskia* Zucc., *Sageratia* Brongn., *Scutia* (DC) Brongn., and *Rhamnus* L. [21,96–99]. The floral disk is often strongly lobed in extant Paliureae, with the sinuses associated with the insertion of the petal-stamen complex; however, it is possible that the size of the lobes in the floral disk changes during development, and, therefore, the absence of lobes in *Notiantha* may not be systematically informative. Based on the suite of characters in the fossil and the various most-parsimonious positions found in the phylogenetic analysis, it is likely that *Notiantha* belongs near the base of the ziziphoid clade.

#### Fossil flowers

The oldest known putative rhamnaceous flowers are the “Rose Creek flowers” from the mid-Cretaceous of Nebraska described by Basinger and Dilcher [100]. Some authors consider these fossils to be the earliest evidence of Rhamnaceae because of the pentamerous, obhaplostemonous arrangement of the floral organs, short-clawed petals, a circular to pentagonal floral disk, a superior ovary, and distinctive pollen sculpture [100,101]. In contrast to crown-group Rhamnaceae, however, the Rose Creek flowers are much larger (20–40 mm across), lack a keel on the sepals, and the stamens are unlike those of extant Rhamnaceae. In the Rose Creek flowers, the filaments are stout, not adnate to the petals, and bear a large basifixed anther, whereas in most extant Rhamnaceae the filaments are slender, adnate to the petals, and bear minute, dorsifixed, and versatile anthers [21]. Burge and Manchester [38] cautioned that petal-opposed stamens also occur in members of Vitales, the apparent sister to all other rosids [102], indicating that this condition might be expected in other early arising rosids. We further suggest, based on the presence of flowers with petal-opposed stamens and/or floral disks in several other groups such as *Quillaja* Molina (Quillajaceae, Fabales), *Dirachma* Schweinf. ex Balf.f. (Dirachmaceae, Rosales) *Lepidobotrys* Engl. (Lepidobotryaceae; Celastrales), and Santalales [95] that the condition of petal-opposed stamens only (obhaplostemony) has repeatedly derived from ancestors with two alternating whorls of stamens (obdiplostemony) in the Pentapetalae. Therefore, until the taxonomic position of the Rose Creek flowers can be confirmed, their placement within crown-group Rhamnaceae is doubtful.

More recently, Late Cretaceous rhamnaceous fossils (flowers, leaves and seeds) were described from the Cerro del Pueblo Formation in Mexico [31] and the Guaduas Formation in Colombia [28]. Among these fossils are the flowers described as *Coahuilanthus belindae* Calvillo-Canadell et Cevallos-Ferriz from El Almácigo locality (Cerro del Pueblo Formation, late Campanian) in General Cepeda County, Coahuila, Mexico (Table 2). *Coahuilanthus* are easily distinguished from *Notiantha* by the petals, which are much shorter than the calyx, and spatulate rather than cucullate as in *Notiantha*. Furthermore, *Coahuilanthus* flowers have a campanulate, rather than obconic, hypanthium and a 10-lobed floral disk. So far, *Coahuilanthus* is the earliest reliable fossil evidence of Rhamnaceae.

**Table 2:** Comparison of fossil flowers assigned to Rhamnaceae.

Taxon	Diam. (mm)	Flower	Floral cup (mm)	Cup shape	Sepal keel	Abaxial sepal surface	Corolla vs calyx	Petal shape	Petal apex	Floral disk	Filament attachment	Anther size	Anthers	Ovary	Locules
*Coahuilanthus belindae* [31]	3–6	Per	2.5–3.5 by 1.1–2.0	Cam	Pres	?	shorter	sp.	R	prominent, 10 lobules at perimeter	unclear	M	?	O	≤4
*Nahinda axamilpensis* [31]	4–8	Per	2.5–5.0 by 1.5–3.5	Cam	Pres	?	shorter	s.c., sp to cu.	N	present, pentagonal perimeter and lobed surface	adnate to petals	M	?	O-O	≤4
*“Solanites” pusillus* [30,103]	8–12	Per	2.0 by 3	Cam to Hem-SD	Pres	H	equal	ob., cu.	R	prominent, circular to pentagonal	?	M	Dv	O	≤4
*Distigouania irregularis* [104]	3–4	Imp	1.2	SD	Pres	G	shorter	sep. & r.l. to ob.	R	prominent, 10-lobed	adnate to petals	M	?	?	≤4
*Comopellis presbya* [105]	4–5	Per	0.8, by 1.7	Hem	Abs	H	shorter	e.l.	N	glabrous, thin, lining hypanthium,	adnate to petals	M	Dv	O	≤4
*Notiantha grandensis*	5–7	Per	1.5–2 by 1.75	Obc	Pres	G	equal	s.c., cu.	N	prominent, pentagonal	adnate to petals	M	Dv	O-O	≤4

Diam. = diameter; Flower: Per = perfect, Imp = imperfect; Cup shape: SD = shallow dish, Cam = campanulate, Hem = hemispherical,

Obc = obconical; Sepal keel: Pres = present, Abs = absent; Abaxial sepal surface: G = glabrous, H = hairy; Petal shape: sp. = spatulate; s.c. = short-clawed; cu. = culcullate; ob. = obovate; sep. = sepaloid; r.l. = rhomboid-lanciolate; e.l. = elliptic-lanciolate. Petal apex: R = rounded, N = notched; Anther size: L = large, M = minute; Anthers: Dv = dorsifixed, versatile; Ovary O = superior, O semi-inferior.

Several genera have been established based on fossilized rhamnaceous flowers from Cenozoic deposits as well, but *Notiantha* may be distinguished from each of these by features of the corolla and the hypanthium (Table 2). The flowers described as *“Solanites” pusillus* Berry from the Eocene of Tennessee, USA [103] were recently recognized as Rhamnaceae by Millán and Crepet [30]. They have a campanulate floral cup and obovate petals each with a rounded apex, features that distinguish *“S*.*” pusillus* from the Patagonian fossils. Comparison of *Notiantha* with the flowers of *Nahinda axamilpensis* Calvillo-Canadell et Cevallos-Ferriz, from the Oligocene of Mexico, reveals that the petals are much shorter in *N*. *axamilpensis* [31]. Chambers and Poinar described two rhamnaceous flowers from Dominican Amber [104,105], *Distigouania irregularis* Chambers et Poinar and *Comopellis presbya* Chambers et Poinar. They compared *D*. *irregularis* with the extant *Gouania* Jacq. and noted the unusual combination of sepaloid petals with a more typical cucullate petal in the same flower. *Notiantha* can easily be distinguished from these two taxa. *D*. *irregularis* is considered a staminate flower, whereas *Notiantha* is perfect. *C*. *presbya* has a wider floral cup than *N*. *grandensis*, cucullate petals that tightly enfold the stamens, and enlarged appendages that radiate from the floral disk. By contrast, the petals of *Notiantha* are clearly short-clawed and do not enfold the anther, and there is no evidence of any kind of enlarged appendages radiating from the floral disk. Clearly, the Patagonian fossil flowers are different from previously described rhamnaceous fossil flowers, and consequently the erection of a new genus and species is warranted.

#### Fossil leaves

Some members of Rhamnaceae produce morphologically distinctive leaves sometimes referred to as rhamnoid- or ziziphoid-type foliage. Leaves of the rhamnoid type are simple, entire, and pinnate, with eucamptodromous secondary veins and closely spaced opposite-percurrent tertiary veins that are nearly perpendicular to the midvein [28,106]. By contrast, leaves of the ziziphoid-type are simple, and palmate with three acrodromous primary veins, mixed alternate-percurrent epimedial tertiary veins, and usually a serrate margin with apically oriented, glandular teeth. Major secondary veins, if present, occur in the distal portion of the leaf blade [51,52]. The rhamnoid and ziziphoid leaf types are distinctive end-members of a range of leaf-types found in Rhamnaceae [28,106,107]. ‘Ziziphoid’ leaves are typical of the Paliureae and some other genera in the ziziphoid clade (e.g. *Ceanothus*, *Colubrina*, *Crumenaria* Mart.), as well as the ampelozizyphoid clade (e.g. *Bathiorhamnus*).

Many of the dispersed rhamnaceous fossil leaves have been assigned to the modern genera *Ziziphus*, *Paliurus*, or *Ceanothus*; however, these extant genera cannot be reliably distinguished based on leaf architecture alone [107], suggesting that the assignment of the fossils to them is dubious. Dispersed leaves that have Rhamnaceae-like morphology but do not fit into the rhamnoid or ziziphoid leaf types are common in the fossil record; however, they are not necessarily identifiable as Rhamnaceae based on venation and margin type alone because genera of other families converge on similar morphology [38,106,108–110]. Even in those cases where an affinity with Rhamnaceae is or could be confirmed, previous assignments to extant genera should be re-evaluated [38,106,107]. Some of the fossil species conforming to the ziziphoid leaf type that should be re-described may be transferred to *Suessenia*.

A thorough review of all fossil “rhamnaceous” leaves is beyond the scope of this work; however, a detailed comparison of *Sussenia* with fossil rhamnaceous leaves reported from South America was performed. The first report of fossil leaves attributed to Rhamnaceae from South America include *Rhamnidium patagonicum* Berry and *R*. *preglabrum* Berry in the Eocene Laguna del Hunco flora [111–113]. These are closer to the rhamnoid leaf type and are unlike *S*. *grandensis*. Later, *Ziziphus chubutensis* Berry, was described based on material collected from the Palacio de los Loros locality in the Salamanca Fm. [15,62,114]. *Z*. *chubutensis* leaves are broadly consistent with those of Paliureae becuase they have three strong basal veins that appear actinodromous (nearly acrodromous), a serrate margin, and glandular teeth. Later, Iglesias et al. [15] suggested that *Banarophyllum ovatum* Berry, also from the Salamanca Fm. and originally allied with Flacourtiaceae, may be a junior synonym of *Z*. *chubutensis* [15,62]. Troncoso [115] reported the occurrence of a single ziziphoid leaf from the Eocene of Chile identified as *Ziziphus* sp.; unfortunately, the fossil is poorly preserved. The base and much of the margin of this specimen are unknown, and therefore this occurrence should be treated with caution. Finally, Correa et al. [28] reported the occurrence of *Berhamniphyllum* from the Late Cretaceous of Colombia, but these also conform to the “rhamnoid” leaf type. *Suessenia* leaves are readily distinguished from those of *Ziziphus chubutensis* because they consistently lack major secondary veins in the distal portion of the blade and because the apex is very acute to attenuate, rather than rounded and obtuse. They are also easily distinguished from the specimen Berry identified as *B*. *ovatum* because they lack major secondary veins, and the tertiary veins are often alternate percurrent, not opposite percurrent. Thus, we consider *Suessenia* distinct from previously reported rhamnaceous leaves from South America and the oldest reliable occurrence of the ziziphoid leaf type.

### Biogeography

Rhamnaceae are distributed throughout tropical and temperate environments worldwide [21,116], but highest diversity is associated with seasonally dry Mediterranean-type environments [29,56]. The broad distribution of the family hindered early efforts to draw conclusions about their biogeographic history [117]. Gentry [55] proposed a Laurasian origin for the family and subsequent expansion into the Southern Hemisphere; however, recent work supports an alternate hypothesis. Richardson et al. and Onstein et al. showed that many of the groups associated with tropical and subtropical forests are early-divergent lineages within Rhamnaceae [26,29,53,56]. Richardson et al. [53] suggested a Gondwanan origin for the family but did not specify the forest type, and Onstein et al. [29,56] emphasized the tropical rainforest aspect of the likely ancestral habitat. The occurrence of fossil Rhamnaceae in the Neotropics during the Late Cretaceous on either side of the Central American Seaway [28,31] is suggestive of an “out of the tropics” scenario for the evolution of the crown-group Rhamnaceae rather than a traditional “Gondwanan” or “Laurasian” origin at mid- or high-latitudes. This hypothesis is consistent with the distribution of several of the extant representatives of the ampelozizyphoids, which are found in Cuba, northern tropical South America, east Africa, and Madagascar, and the primarily Neogene radiations of more derived rhamnoid and ziziphoid lineages in Mediterranean habitats worldwide [23,29,56,87].

There are 16 extant genera of Rhamnaceae native to the southern cone of South America (Chile, Argentina, Paraguay, Uruguay, and southern Brazil), and one of them is endemic to Chile [118–120]. Much of the species richness in that region, particularly in the Colletieae Reissek ex Endl. [86], is found in the Mediterranean-type climate of Chile and western Argentina, whereas other lineages, including *Sarcomphalus* (formerly new-world *Ziziphus*), *Hovenia* (introduced), *Colubrina*, and *Gouania* Jacq. are found in the subtropical forests of northern Argentina [118,119].

Southern Chubut Province (Argentina), where the fossils were collected, has a semi-arid to cold-steppe ecoregion [121]. Today, in the San Jorge Basin, the mean annual temperature is 11.5°C and mean annual precipitation is 16.4 cm yr^-1^ [122]. *Condalia*, *Colletia* Comm. Ex Juss., *Discaria* Hook., and *Trevoa* Miers can be found in the region today (pers. obs.). By contrast, paleoclimate estimates for the Salamanca Fm. biota suggest subtropical lowland environment with a mean annual temperature of ~13–14°C, and mean annual rainfall of 115–124 cm yr^-1^ based on foliar physiognomy [82] and the presence of thermophilic groups (palms, podocarps, and alligatorids) [123]. This reconstructed climate is similar to the subtropical forests of northern Argentina where *Sarcomphalus*, *Hovenia*, and *Colubrina* grow today [29].

Most fossils assigned to Rhamnaceae have been collected from Eocene and younger deposits across the Northern Hemisphere (Fig 1; Table 1). Although the fossils described by Correa et al. [28] are from South America, they are not technically from the Southern Hemisphere. The only previous reports of rhamnaceous fossils from the Southern Hemisphere were based on dispersed leaves [62,111,115,124,125] or pollen [126–130]. Two of these pollen occurrences are from Antarctica, the only continent where Rhamnaceae does not grow today (Fig 1). Fossil wood [131,132] and fruits [133,134] attributed to Rhamnacae have been reported from the Deccan Intertrappean beds of India, which was in the Southern Hemisphere or straddled the equator for much of the Late Cretaceous and Paleogene; however, Guleria [135] and Prakash [136] found that these records are unreliable. The fossils either do not preserve some of the features that are necessary to confirm or reject the rhamnaceous affinities, or they show closer affinities with other families. The remarkably sparse fossil record of Rhamnaceae from the Southern Hemisphere contrasts with the rich record in the Northern Hemisphere. Traditionally, this has been thought to reflect a Laurasian origin for the family and subsequent expansion into Gondwanan landmasses [137]; however, a simpler explanation may be that geographical sampling bias drives this pattern. Recent work has demonstrated the potential for new discoveries in the Southern Hemisphere to improve our understanding of the history of various widespread (or formerly widespread) groups [28,138–142].

### Conclusion

The fossils described here are, to our knowledge, the first early Danian flowers known from the Southern Hemisphere. They are also the southernmost fossil occurrence of Rhamnaceae flowers and the only unequivocal megafossil occurrence of the family in the Southern Hemisphere. Based on the results of our phylogenetic analyses together with all available evidence, we argue that the discovery of *Notiantha* provides a reliable minimum age of 66 Million years for the node that unites the extant ziziphoids; the most conservative approach would be to apply a minimum age of 66 Million years for the node that unites the ziziphoid and ampeloziziphoid clade, i.e. the base of the crown-group. The Late Cretaceous occurrences of Rhamnaceae from the Neotropics and the tropical distribution of several extant, early-diverging lineages of the family indicates that the initial diversification of the family took place in warm and wet tropical to subtropical forests, and the family later spread to temperate and Mediterranean biomes [56]. The discovery of *Notiantha* (flowers) together with *Suessenia* (ziziphoid leaves) from the Salamanca Formation confirms that Rhamnaceae reached southern South America by the early Paleocene and raises the possibility for southern dispersal routes via Antarctica and subsequent vicariance [143–147] to help explain biogeographic patterns of Rhamnaceae [53].

## Supporting information

S1 Table Comparative material of extant Rhamnaceae. List of examined comparative material of extant Rhamnaceae. US: United States National Herbarium; NCLC-H: National Cleared Leaf Collection-Hickey; FLAS: University of Florida Herbarium; BH: Bailey Hortorium, Cornell University.
(DOCX)

S1 Fig Hauenschild et al. topology including floral and foliar characters. Phylogeny including One of five equally most parsimonious trees based on floral and foliar characters and the topology of Hauenschild et al. [23] showing the position of *Notiantha* sister to the extant Paliureae [*Hovenia*+*Sarcomphalus*+*Paliurus*+*Ziziphus*] at arrow. The four alternate most parsimonious positions for the fossil flowers in the ziziphoid clade are colored in dark green. A = Ampelozizypheae, B = Bathiorhamneae, V = Ventilagineae, R = Rhamneae, Po = Pomaderreae, C = Colletieae, Ph = Phyliceae, U = unplaced genera at tribal level, G = Gouanieae, Pa = Paliureae.
(TIF)

S2 Fig Onstein et al. topology including floral characters. One of eight equally most parsimonious trees based on floral characters the topology of Onstein et al. [29] showing the position of *Notiantha* nested in Paliureae sister to [*Hovenia*+*Paliurus*+*Ziziphus*] at arrow. The seven alternate most parsimonious positions for the fossil flowers are colored in dark green. A = Ampelozizypheae, B = Bathiorhamneae, V = Ventilagineae, R = Rhamneae, Po = Pomaderreae, C = Colletieae, Ph = Phyliceae, U = unplaced genera at tribal level, G = Gouanieae, Pa = Paliureae. This result was obtained using only the first 25 floral characters and is therefore conservative.
(TIF)

S3 Fig Onstein et al. topology including floral and foliar characters. One of four equally most parsimonious trees based on floral and foliar characters and the topology of Onstein et al. [29] showing the position of *Notiantha* nested in Paliureae sister to [*Hovenia*+*Paliurus*+*Ziziphus*] at arrow. The three alternate most parsimonious positions for the fossil flowers are colored in dark green. A = Ampelozizypheae, B = Bathiorhamneae, V = Ventilagineae, R = Rhamneae, Po = Pomaderreae, C = Colletieae, Ph = Phyliceae, U = unplaced genera at tribal level, G = Gouanieae, Pa = Paliureae.
(TIF)
