# Supplementary material for: Flowering after disaster: Early Danian buckthorn (Rhamnaceae) flowers and leaves from Patagonia
Source: PLoS One. 2017 May 10;12(5):e0176164. doi: 10.1371/journal.pone.0176164 (PMC5425202; doi:10.1371/journal.pone.0176164)
Supplement: S1 Table — List of examined comparative material of extant Rhamnaceae. US: United States National Herbarium; NCLC-H: National Cleared Leaf Collection-Hickey; FLAS: University of Florida Herbarium; BH: Bailey Hortorium, Cornell University. (DOCX) [file pone.0176164.s001.docx]

| Species | Herbarium number |
| --- | --- |
| *Paliurus aculeatus* | US 136512 |
| *Paliurus spina-christi* | US 1575969 |
| *Sarcomphalus mistol* | US 1904580 |
| *Sarcomphalus mistol* | US 1904704 |
| *Sarcomphalus saeri* | US 1708023 |
| *Sarcomphalus saeri* | US 2045934 |
| *Sarcomphalus saeri* | US 3554997 |
| *Sarcomphalus saeri* | US 2121459 |
| *Zizophus inermis* | NCLC-H 1794 |
| *Ziziphus sativa* | NCLC-H 1791 |
| *Ceanothus americanus* | FLAS 68528 |
| *Colubrina arborescens* | FLAS 233725 |
| *Colubrina arborescens* | FLAS 39146 |
| *Gouania lupuloides* | FLAS 25704 |
| *Gouania lupuloides* | FLAS 139050 |
| *Krugiodendron ferreum* | FLAS 188080 |
| *Krugiodendron ferreum* | FLAS 23224 |
| *Paliurus ramosissus* | FLAS 52107 |
| *Pomaderris apetala* | FLAS 62010 |
| *Pomaderris elliptica* | FLAS 62011 |
| *Reynosia septentrionalis* | FLAS 238058 |
| *Rhamnus caroliniana* | FLAS 170324 |
| *Rhamnus caroliniana* | FLAS 141423 |
| *Sageretia minutiflora* | FLAS 165577 |
| *Spyridium parvifolium* | FLAS 162178 |
| *Trymalium ledifolium* | FLAS 162179 |
| *Ventilago calyculata* | FLAS 135783 |
| *Ziziphus zizyphus* | FLAS 9170 |
| *Adolphia californica* | BH 115038 |
| *Adolphia californica* | BH 115042 |
| *Adolphia californica* | BH 115043 |
| *Adolphia infesta* | BH 115039 |
| *Adolphia infesta* | BH 115040 |
| *Adolphia infesta* | BH 115041 |
| *Alphitonia ponderosa* | BH 114883 |
| *Alphitonia ponderosa* | BH 114884 |
| *Alphitonia ponderosa* | BH 115049 |
| *Alphitonia ponderosa* | BH 115050 |
| *Alphitonia ponderosa* | BH 115051 |
| *Alphitonia ponderosa* | BH 115052 |
| *Alphitonia ponderosa* | BH 115053 |
| *Alphitonia ponderosa* | BH 115054 |
| *Alphitonia ponderosa* | BH 115055 |
| *Alphitonia ponderosa* | BH 115055 |
| *Alphitonia ponderosa* | BH 115056 |
| *Alphitonia zizyphoides* | BH 115044 |
| *Alphitonia zizyphoides* | BH 115045 |
| *Alphitonia zizyphoides* | BH 115046 |
| *Alphitonia zizyphoides* | BH 115047 |
| *Alphitonia zizyphoides* | BH 115047 |
| *Alphitonia zizyphoides* | BH 115048 |
| *Berchemia scandens* | BH 115123 |
| *Berchemia scandens* | BH 115124 |
| *Berchemia scandens* | BH 115125 |
| *Berchemia scandens* | BH 115126 |
| *Berchemia scandens* | BH 115127 |
| *Berchemia scandens* | BH 115128 |
| *Berchemia scandens* | BH 115129 |
| *Berchemia scandens* | BH 115130 |
| *Berchemia scandens* | BH 115131 |
| *Berchemia scandens* | BH 115132 |
| *Berchemia scandens* | BH 115133 |
| *Berchemia scandens* | BH 115134 |
| *Berchemia scandens* | BH 115135 |
| *Berchemia scandens* | BH 115136 |
| *Berchemia scandens* | BH 115137 |
| *Ceanothus americanus* | BH 115492 |
| *Ceanothus americanus* | BH 115497 |
| *Ceanothus americanus* | BH 115498 |
| *Ceanothus americanus* | BH 115499 |
| *Ceanothus americanus* | BH 115500 |
| *Ceanothus americanus* | BH 115501 |
| *Ceanothus americanus* | BH 115502 |
| *Ceanothus americanus* | BH 115503 |
| *Ceanothus americanus* | BH 115504 |
| *Ceanothus americanus* | BH 115505 |
| *Ceanothus americanus* | BH 115506 |
| *Ceanothus americanus* | BH 115507 |
| *Ceanothus americanus* | BH 115508 |
| *Ceanothus americanus* | BH 115509 |
| *Ceanothus americanus* | BH 115510 |
| *Ceanothus americanus* | BH 115511 |
| *Ceanothus americanus* | BH 115512 |
| *Colubrina asiatica* | BH 118294 |
| *Colubrina asiatica* | BH 118295 |
| *Colubrina cubensis* | BH 115600 |
| *Colubrina ferruginea* | BH 115599 |
| *Colubrina glabra* | BH 115601 |
| *Colubrina glabra* | BH 115602 |
| *Colubrina glandulosa* | BH 115604 |
| *Colubrina glomerata* | BH 115597 |
| *Colubrina greggii* | BH 115596 |
| *Colubrina greggii* | BH 115598 |
| *Colubrina greggii* | BH 115603 |
| *Colubrina greggii* | BH 115605 |
| *Colubrina greggii* | BH 115606 |
| *Colubrina macrocarpa* | BH 115586 |
| *Colubrina megacarpa* | BH 115585 |
| *Colubrina megacarpa* | BH 115588 |
| *Colubrina montana* | BH 115589 |
| *Colubrina reclinata* | BH 115590 |
| *Colubrina reclinata* | BH 115591 |
| *Colubrina reclinata* | BH 115592 |
| *Colubrina retusa* | BH 115594 |
| *Colubrina rufa* | BH 115587 |
| *Colubrina sp.* | BH 115593 |
| *Colubrina spinosa* | BH 115595 |
| *Condalia lycioides* | BH 115730 |
| *Condalia lycioides* | BH 115731 |
| *Condalia lycioides* | BH 115732 |
| *Condalia obovata* | BH 115733 |
| *Condalia obovata* | BH 115734 |
| *Condalia obovata* | BH 116777 |
| *Condalia obovata* | BH 116778 |
| *Fragaria virgata* | BH 116799 |
| *Fragaria virginiana* | BH 116236 |
| *Frangula alnus* | BH 115755 |
| *Frangula frangula* | BH 115754 |
| *Frangula vulgaris* | BH 115752 |
| *Frangula vulgaris* | BH 115753 |
| *Gouania longipetala* | BH 118296 |
| *Gouania longispicata* | BH 118297 |
| *Gouania polygama* | BH 116852 |
| *Gouania polygama* | BH 116853 |
| *Gouania polygama* | BH 116854 |
| *Gouania polygama* | BH 116855 |
| *Gouania polygama* | BH 116856 |
| *Gouania polygama* | BH 116857 |
| *Gouania polygama* | BH 116858 |
| *Gouania polygama* | BH 116859 |
| *Gouania scandens* | BH 118298 |
| *Helinus integrifolius* | BH 116842 |
| *Helinus integrifolius* | BH 116843 |
| *Helinus integrifolius* | BH 116844 |
| *Helinus integrifolius* | BH 116845 |
| *Helinus scandens* | BH 116846 |
| *Helinus spartioides* | BH 116847 |
| *Helinus spartioides* | BH 116848 |
| *Hovenia dulcis* | BH 116849 |
| *Hovenia dulcis* | BH 116850 |
| *Hovenia dulcis* | BH 116851 |
| *Karwinska humboldtiana* | BH 116860 |
| *Karwinska humboldtiana* | BH 116861 |
| *Karwinska humboldtiana* | BH 116862 |
| *Karwinska humboldtiana* | BH 116863 |
| *Karwinska humboldtiana* | BH 116864 |
| *Karwinska humboldtiana* | BH 116865 |
| *Karwinska humboldtiana* | BH 116866 |
| *Karwinska humboldtiana* | BH 116867 |
| *Karwinska humboldtiana* | BH 116868 |
| *Karwinska humboldtiana* | BH 116869 |
| *Karwinska humboldtiana* | BH 116870 |
| *Karwinska humboldtiana* | BH 116871 |
| *Karwinska humboldtiana* | BH 116872 |
| *Karwinskia humboldtiana* | BH 116873 |
| *Karwinskia humboldtiana* | BH 116874 |
| *Karwinskia humboldtiana* | BH 116875 |
| *Karwinskia humboldtiana* | BH 116876 |
| *Karwinskia humboldtiana* | BH 116877 |
| *Karwinskia humboldtiana* | BH 116878 |
| *Karwinskia humboldtiana* | BH 116879 |
| *Karwinskia humboldtiana* | BH 116880 |
| *Karwinskia humboldtiana* | BH 116881 |
| *Karwinskia humboldtiana* | BH 116882 |
| *Krugiodendron ferreum* | BH 116883 |
| *Krugiodendron ferreum* | BH 116884 |
| *Krugiodendron ferreum* | BH 116885 |
| *Krugiodendron ferreum* | BH 116886 |
| *Krugiodendron ferreum* | BH 116887 |
| *Krugiodendron ferreum* | BH 116888 |
| *Krugiodendron ferreum* | BH 116889 |
| *Krugiodendron ferreum* | BH 116890 |
| *Krugiodendron ferreum* | BH 116891 |
| *Noltea africana* | BH 116892 |
| *Noltea africana* | BH 116893 |
| *Paliurus aculeatus* | BH 118334 |
| *Paliurus aculeatus* | BH 118342 |
| *Paliurus aculeatus* | BH 118343 |
| *Paliurus australis* | BH 118457 |
| *Paliurus australis* | BH 118478 |
| *Paliurus orientalis* | BH 118340 |
| *Paliurus paliurus* | BH 118455 |
| *Paliurus ramosissimus* | BH 118335 |
| *Paliurus ramosissimus* | BH 118336 |
| *Paliurus ramosissimus* | BH 118461 |
| *Paliurus spina-christi* | BH 118337 |
| *Paliurus spina-christi* | BH 118339 |
| *Paliurus spina-christi* | BH 118341 |
| *Paliurus spina-christi* | BH 118344 |
| *Paliurus spina-christi* | BH 118456 |
| *Paliurus spina-christi* | BH 118458 |
| *Paliurus spina-christi* | BH 118459 |
| *Paliurus spina-christi* | BH 118460 |
| *Paliurus spina-christi* | BH 118462 |
| *Paliurus spina-christi* | BH 118463 |
| *Paliurus spina-christi* | BH 118464 |
| *Paliurus spina-christi* | BH 118465 |
| *Paliurus spina-christi* | BH 118466 |
| *Paliurus spina-christi* | BH 118479 |
| *Phylica aemula* | BH 118422 |
| *Phylica ambigua* | BH 118410 |
| *Phylica ambigua* | BH 118411 |
| *Phylica axillaris* | BH 118299 |
| *Phylica axillaris* | BH 118300 |
| *Phylica axillaris* | BH 118301 |
| *Phylica axillaris* | BH 118412 |
| *Phylica axillaris* | BH 118413 |
| *Phylica buxifolia* | BH 118409 |
| *Phylica capitata* | BH 118408 |
| *Phylica cephalantha* | BH 118407 |
| *Phylica cryptandroides* | BH 118405 |
| *Phylica cryptandroides* | BH 118406 |
| *Phylica cuspidata* | BH 118404 |
| *Phylica ericoides* | BH 116894 |
| *Phylica ericoides* | BH 116895 |
| *Phylica ericoides* | BH 116896 |
| *Phylica ericoides* | BH 116897 |
| *Phylica eriophoros* | BH 118403 |
| *Phylica excelsa* | BH 118401 |
| *Phylica floribunda* | BH 118390 |
| *Phylica gnidioides* | BH 118391 |
| *Phylica gnidioides* | BH 118393 |
| *Phylica hirta* | BH 118394 |
| *Phylica imberbis* | BH 118395 |
| *Phylica imberbis* | BH 118396 |
| *Phylica lasicarpa* | BH 118398 |
| *Phylica lechneaeoides* | BH 118397 |
| *Phylica marlothii* | BH 118399 |
| *Phylica nervosa* | BH 118400 |
| *Phylica nigrita* | BH 118389 |
| *Phylica odorata* | BH 118388 |
| *Phylica paniculata* | BH 118414 |
| *Phylica paniculata* | BH 118415 |
| *Phylica paniculata* | BH 118416 |
| *Phylica paniculata* | BH 118417 |
| *Phylica parviflora* | BH 118386 |
| *Phylica parviflora* | BH 118387 |
| *Phylica pinea* | BH 118385 |
| *Phylica plumosa* | BH 118368 |
| *Phylica plumosa* | BH 118379 |
| *Phylica plumosa* | BH 118380 |
| *Phylica pubescens* | BH 118383 |
| *Phylica pubescens* | BH 118384 |
| *Phylica purpurea* | BH 118382 |
| *Phylica pustulata* | BH 118381 |
| *Phylica rigida* | BH 118370 |
| *Phylica selaginoides* | BH 118369 |
| *Phylica selaginoides* | BH 118371 |
| *Phylica sp.* | BH 118366 |
| *Phylica spicata* | BH 118372 |
| *Phylica spicata* | BH 118373 |
| *Phylica stipularia* | BH 118418 |
| *Phylica stipularia* | BH 118419 |
| *Phylica stipularis* | BH 118420 |
| *Phylica stipularis* | BH 118421 |
| *Phylica strigulosa* | BH 118374 |
| *Phylica verticellata* | BH 118375 |
| *Phylica virginata* | BH 118376 |
| *Phylica willdenowiana* | BH 118367 |
| *Phylica willdenowiana* | BH 118377 |
| *Phylica willdenowiana* | BH 118378 |
| *Pomaderris edgerleyi* | BH 116900 |
| *Pomaderris edgerleyi* | BH 116901 |
| *Pomaderris elliptica* | BH 116899 |
| *Pomaderris kumeraho* | BH 116902 |
| *Pomaderris phylicaefolia* | BH 116903 |
| *Pomaderris rugosa* | BH 116904 |
| *Pomaderris sp.* | BH 116898 |
| *Reynosia latifolia* | BH 118480 |
| *Reynosia latifolia* | BH 118481 |
| *Reynosia septentrionalis* | BH 118482 |
| *Reynosia septrionalis* | BH 118483 |
| *Reynosia septrionalis* | BH 118484 |
| *Reynosia septrionalis* | BH 118485 |
| *Reynosia septrionalis* | BH 118489 |
| *Rhamnus obliqua* | BH 118795 |
| *Rhamnus pompana* | BH 118794 |
| *Rhamnus pringlei* | BH 118793 |
| *Rhamnus prinoides* | BH 118357 |
| *Rhamnus prinoides* | BH 118358 |
| *Rhamnus prinoides* | BH 118359 |
| *Rhamnus prinoides* | BH 118360 |
| *Rhamnus prinoides* | BH 118361 |
| *Rhamnus prinoides* | BH 118363 |
| *Rhamnus prinoides* | BH 118365 |
| *Rhamnus prunioides* | BH 118364 |
| *Rhamnus prunoides* | BH 118362 |
| *Rhamnus saxatilis* | BH 118792 |
| *Rhamnus serrata* | BH 118790 |
| *Rhamnus serrata* | BH 118791 |
| *Rhamnus sharpii* | BH 118789 |
| *Sageretia elegans* | BH 117848 |
| *Sageretia elegans* | BH 117849 |
| *Sageretia elegans* | BH 117850 |
| *Sageretia michauxii* | BH 117845 |
| *Sageretia minutiflora* | BH 117844 |
| *Sageretia minutiflora* | BH 117846 |
| *Sageretia minutiflora* | BH 117847 |
| *Sarcomphalus mauritana* | BH 117842 |
| *Sarcomphalus mauritania* | BH 117843 |
| *Sarcomphalus mauritiana* | BH 118145 |
| *Sarcomphalus mauritiana* | BH 117829 |
| *Sarcomphalus mauritiana* | BH 117840 |
| *Sarcomphalus mauritiana* | BH 118142 |
| *Sarcomphalus mauritiana* | BH 118143 |
| *Sarcomphalus mauritiana* | BH 118144 |
| *Sarcomphalus mauritiana* | BH 117841 |
| *Sarcomphalus mauritiana* | BH 117987 |
| *Sarcomphalus mauritiana* | BH 117988 |
| *Sarcomphalus mauritiana* | BH 117989 |
| *Sarcomphalus mauritiana* | BH 118146 |
| *Sarcomphalus mauritiana* | BH 118147 |
| *Sarcomphalus mauritiana* | BH 118148 |
| *Sarcomphalus mauritiana* | BH 118149 |
| *Sarcomphalus mauritianus* | BH 118451 |
| *Sarcomphalus mistol* | BH 117832 |
| *Sarcomphalus mistol* | BH 117833 |
| *Sarcomphalus mistol* | BH 117830 |
| *Sarcomphalus mistol* | BH 117831 |
| *Sarcomphalus sativa* | BH 118349 |
| *Sarcomphalus sativa* | BH 118350 |
| *Sarcomphalus sonorensis* | BH 117578 |
| *Sarcomphalus sonorensis* | BH 117585 |
| *Sarcomphalus sonorensis* | BH 117584 |
| *Scutia myrtica* | BH 118435 |
| *Scutia myrtina* | BH 118423 |
| *Scutia myrtina* | BH 118424 |
| *Scutia myrtina* | BH 118425 |
| *Scutia myrtina* | BH 118426 |
| *Scutia myrtina* | BH 118427 |
| *Scutia myrtina* | BH 118428 |
| *Scutia myrtina* | BH 118429 |
| *Scutia myrtina* | BH 118430 |
| *Scutia myrtina* | BH 118431 |
| *Scutia myrtina* | BH 118432 |
| *Scutia myrtina* | BH 118433 |
| *Scutia myrtina* | BH 118434 |
| *Ziziphus abyssinica* | BH 118450 |
| *Ziziphus abyssinica* | BH 118756 |
| *Ziziphus joazeiro* | BH 117985 |
| *Ziziphus jujuba* | BH 117569 |
| *Ziziphus jujuba* | BH 117570 |
| *Ziziphus jujuba* | BH 117574 |
| *Ziziphus jujuba* | BH 117575 |
| *Ziziphus jujuba* | BH 118122 |
| *Ziziphus jujuba* | BH 118126 |
| *Ziziphus jujuba* | BH 118127 |
| *Ziziphus jujuba* | BH 118128 |
| *Ziziphus jujuba* | BH 118130 |
| *Ziziphus jujuba* | BH 118131 |
| *Ziziphus jujuba* | BH 117565 |
| *Ziziphus jujuba* | BH 117566 |
| *Ziziphus jujuba* | BH 117567 |
| *Ziziphus jujuba* | BH 117568 |
| *Ziziphus jujuba* | BH 117571 |
| *Ziziphus jujuba* | BH 117572 |
| *Ziziphus jujuba* | BH 117573 |
| *Ziziphus jujuba* | BH 117961 |
| *Ziziphus jujuba* | BH 117962 |
| *Ziziphus jujuba* | BH 117963 |
| *Ziziphus jujuba* | BH 117964 |
| *Ziziphus jujuba* | BH 117965 |
| *Ziziphus jujuba* | BH 117983 |
| *Ziziphus jujuba* | BH 117990 |
| *Ziziphus jujuba* | BH 117991 |
| *Ziziphus jujuba* | BH 118123 |
| *Ziziphus jujuba* | BH 118124 |
| *Ziziphus jujuba* | BH 118125 |
| *Ziziphus jujuba* | BH 118129 |
| *Ziziphus jujuba* | BH 118132 |
| *Ziziphus jujuba* | BH 118133 |
| *Ziziphus jujuba* | BH 118134 |
| *Ziziphus jujuba* | BH 118135 |
| *Ziziphus lotus* | BH 115120 |
| *Ziziphus lotus* | BH 118345 |
| *Ziziphus lotus* | BH 118346 |
| *Ziziphus lycioides* | BH 117992 |
| *Ziziphus lycioides* | BH 117993 |
| *Ziziphus mucronata* | BH 118136 |
| *Ziziphus mucronata* | BH 118141 |
| *Ziziphus mucronata* | BH 117999 |
| *Ziziphus mucronata* | BH 118137 |
| *Ziziphus mucronata* | BH 118140 |
| *Ziziphus mucronata* | BH 118436 |
| *Ziziphus mucronata* | BH 118437 |
| *Ziziphus mucronata* | BH 118438 |
| *Ziziphus mucronata* | BH 118439 |
| *Ziziphus mucronata* | BH 118440 |
| *Ziziphus mucronata* | BH 118441 |
| *Ziziphus mucronata* | BH 118442 |
| *Ziziphus mucronata* | BH 118443 |
| *Ziziphus mucronata* | BH 118444 |
| *Ziziphus mucronata* | BH 118445 |
| *Ziziphus mucronata* | BH 118446 |
| *Ziziphus mucronata* | BH 118447 |
| *Ziziphus mucronata* | BH 118448 |
| *Ziziphus mucronata* | BH 118449 |
| *Ziziphus mucronata* | BH 118763 |
| *Ziziphus pedunculata* | BH 117579 |
| *Ziziphus pubescens* | BH 117994 |
| *Ziziphus pubescens* | BH 117995 |
| *Ziziphus pubescens* | BH 117996 |
| *Ziziphus pubescens* | BH 117997 |
| *Ziziphus reticulata* | BH 117580 |
| *Ziziphus reticulata* | BH 117581 |
| *Ziziphus rignonii* | BH 117582 |
| *Ziziphus ringonii* | BH 117583 |
| *Ziziphus sp.* | BH 117576 |
| *Ziziphus sp.* | BH 118452 |
| *Ziziphus spina-christi* | BH 118139 |
| *Ziziphus spina-christi* | BH 117577 |
| *Ziziphus spina-christi* | BH 118338 |
| *Ziziphus vulgaris* | BH 118348 |
| *Ziziphus vulgaris* | BH 117984 |
| *Ziziphus vulgaris* | BH 118347 |
| *Ziziphus zizyphus* | BH 118138 |
