# Supplementary figures and images for: Flowering after disaster: Early Danian buckthorn (Rhamnaceae) flowers and leaves from Patagonia
Source: PLoS One. 2017 May 10;12(5):e0176164. doi: 10.1371/journal.pone.0176164 (PMC5425202; doi:10.1371/journal.pone.0176164)

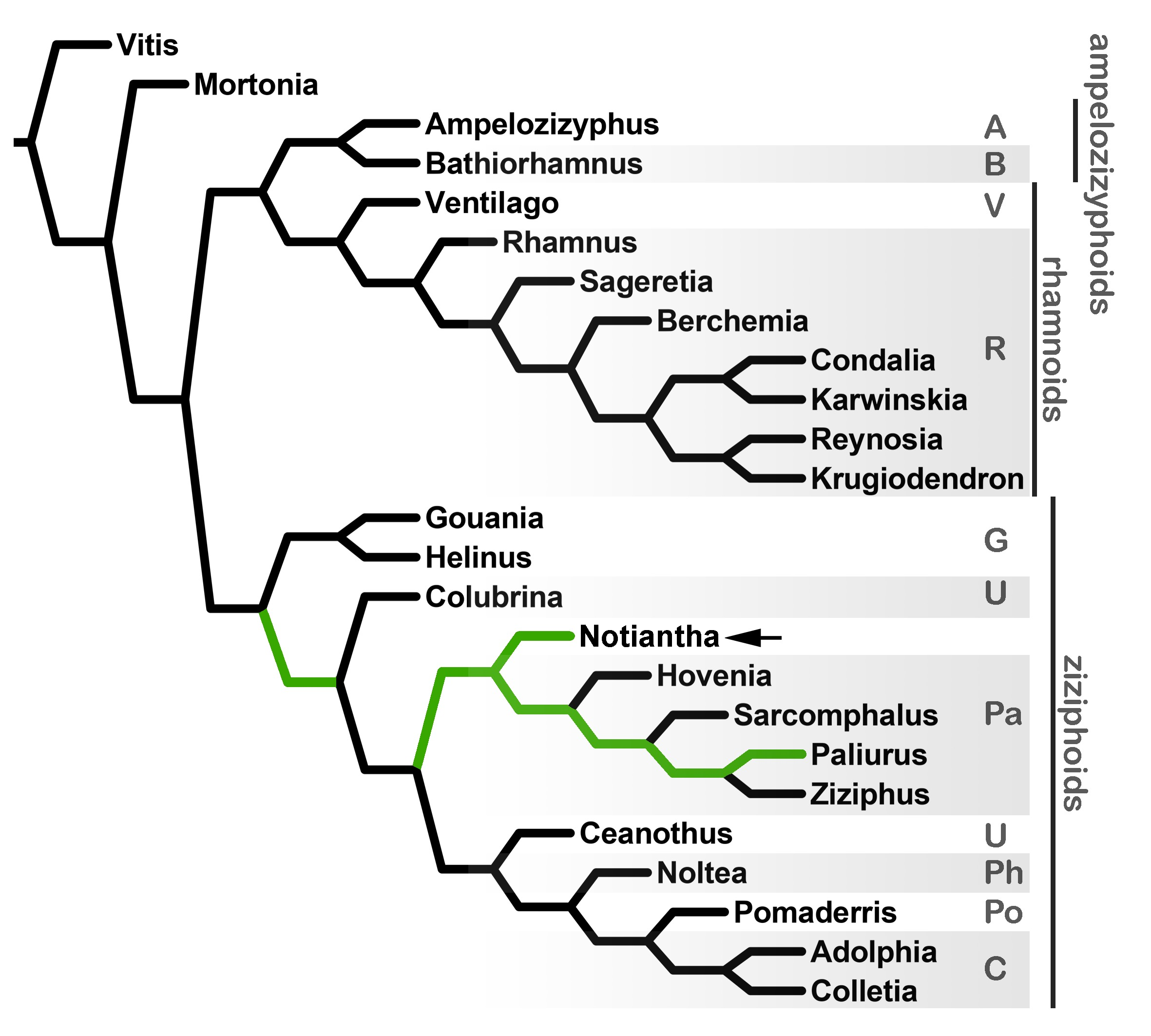

Supplement: S1 Fig — Phylogeny including One of five equally most parsimonious trees based on floral and foliar characters and the topology of Hauenschild et al. [23] showing the position of Notiantha sister to the extant Paliureae [Hovenia+Sarcomphalus+Paliurus+Ziziphus] at arrow. The four alternate most parsimonious positions for the fossil flowers in the ziziphoid clade are colored in dark green. A = Ampelozizypheae, B = Bathiorhamneae, V = Ventilagineae, R = Rhamneae, Po = Pomaderreae, C = Colletieae, Ph = Phyliceae, U = unplaced genera at tribal level, G = Gouanieae, Pa = Paliureae. (TIF) [file pone.0176164.s002.tif]

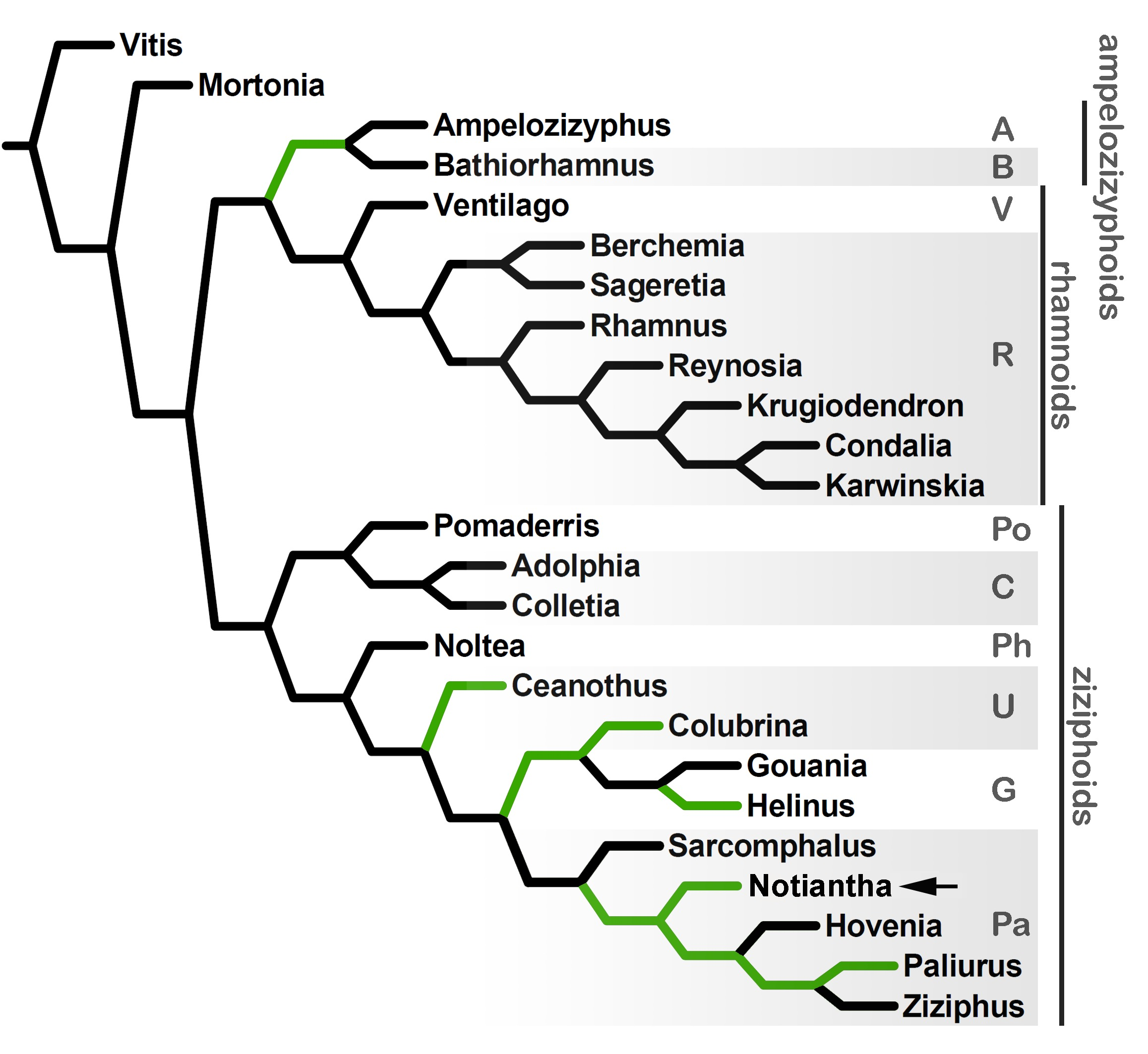

Supplement: S2 Fig — One of eight equally most parsimonious trees based on floral characters the topology of Onstein et al. [29] showing the position of Notiantha nested in Paliureae sister to [Hovenia+Paliurus+Ziziphus] at arrow. The seven alternate most parsimonious positions for the fossil flowers are colored in dark green. A = Ampelozizypheae, B = Bathiorhamneae, V = Ventilagineae, R = Rhamneae, Po = Pomaderreae, C = Colletieae, Ph = Phyliceae, U = unplaced genera at tribal level, G = Gouanieae, Pa = Paliureae. This result was obtained using only the first 25 floral characters and is therefore conservative. (TIF) [file pone.0176164.s003.tif]

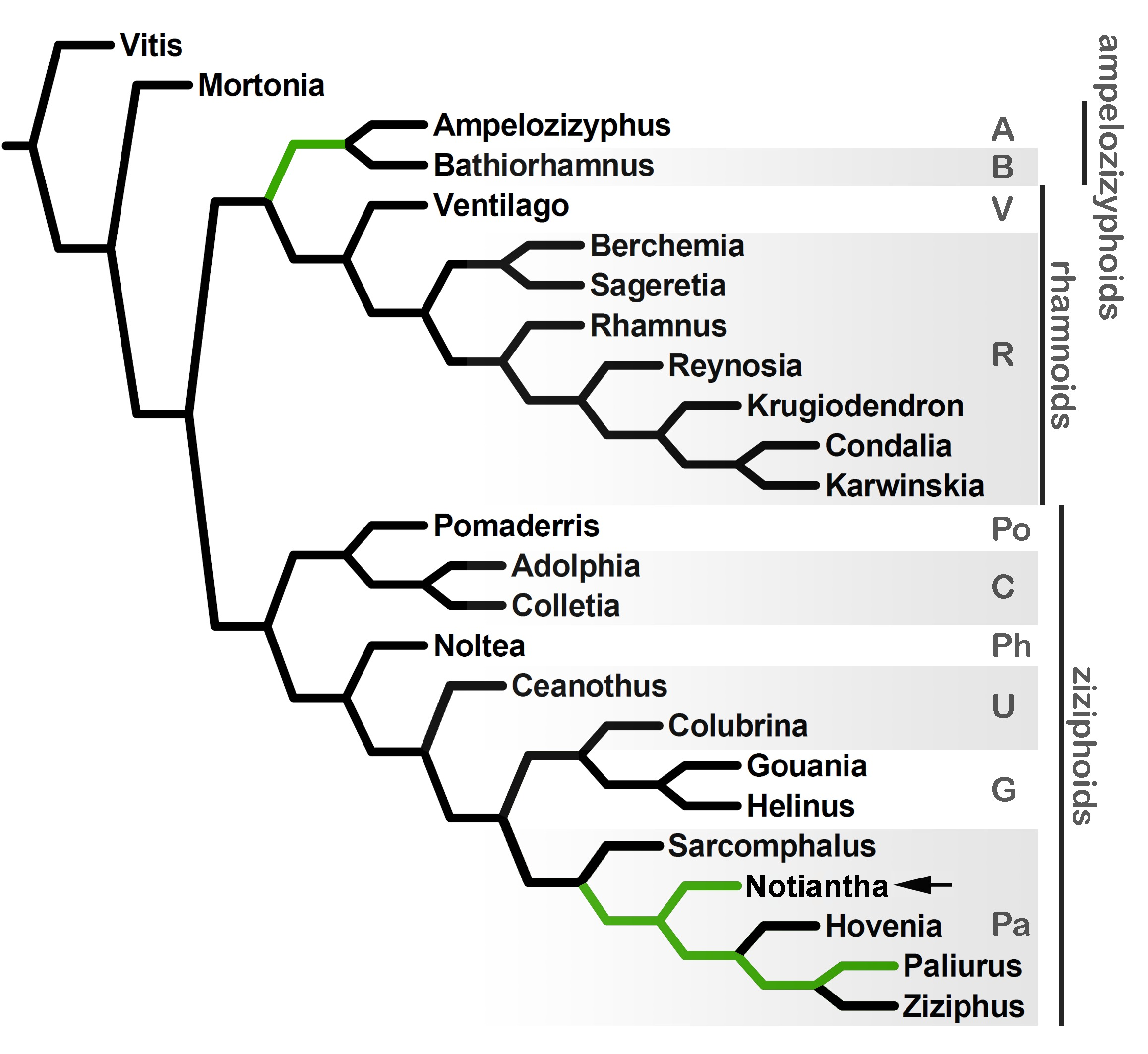

Supplement: S3 Fig — One of four equally most parsimonious trees based on floral and foliar characters and the topology of Onstein et al. [29] showing the position of Notiantha nested in Paliureae sister to [Hovenia+Paliurus+Ziziphus] at arrow. The three alternate most parsimonious positions for the fossil flowers are colored in dark green. A = Ampelozizypheae, B = Bathiorhamneae, V = Ventilagineae, R = Rhamneae, Po = Pomaderreae, C = Colletieae, Ph = Phyliceae, U = unplaced genera at tribal level, G = Gouanieae, Pa = Paliureae. (TIF) [file pone.0176164.s004.tif]
